# Comparative proteomic analysis of the effect of temperature and fertilizer on gliadin and glutenin accumulation in the developing endosperm and flour from *Triticum aestivum* L. cv. Butte 86

**DOI:** 10.1186/1477-5956-11-8

**Published:** 2013-02-22

**Authors:** William J Hurkman, Charlene K Tanaka, William H Vensel, Roger Thilmony, Susan B Altenbach

**Affiliations:** 1 U.S. Department of Agriculture, Agricultural Research Service, Western Regional Research Center, 800 Buchanan St, Albany, CA, 94710, USA

**Keywords:** Endosperm, Fertilizer, Flour, Gliadins, Glutenins, Proteome, Temperature, Wheat

## Abstract

**Background:**

Flour quality is largely determined by the gluten proteins, a complex mixture of proteins consisting of high molecular weight-glutenin subunits (HMW-GS), low molecular weight-glutenin subunits (LMW-GS), and α-, γ-, and ω-gliadins. Detailed proteomic analyses of the effects of fertilizer and high temperature on individual gliadin and glutenin protein levels are needed to determine how these environmental factors influence flour quality.

**Results:**

Wheat plants (*Triticum aestivum* L. cv. Butte 86) were grown in greenhouses under moderate and high temperature regimens with and without post-anthesis fertilizer. Quantitative two-dimensional gel electrophoresis was used to construct accumulation profiles in developing endosperm for the entire complement of gluten proteins identified previously by tandem mass spectrometry. Amounts of individual gliadins and glutenins were also determined in flour produced under each of the regimens. Under all environmental regimens, most HMW-GS, LMW-GS, γ- and ω-gliadins accumulated rapidly during early stages of grain development and leveled off during middle stages of development. A subset of LMW-GS showed a second distinct profile, accumulating throughout development, while α-gliadins showed a variety of accumulation profiles. In flour, fourteen distinct gluten proteins responded similarly to fertilizer, high temperature, and high temperature plus fertilizer. The majority of HMW-GS and ω-gliadins and some α-gliadins increased while two LMW-GS and a minor γ-gliadin decreased. Fertilizer did not influence gluten protein accumulation under high temperature conditions. Additionally, the effects of fertilizer and high temperature were not additive; very few changes were observed when plants that received fertilizer were subjected to high temperature.

**Conclusions:**

Although post-anthesis temperature and fertilizer have very different effects on grain development and yield, the two treatments elicit surprisingly similar effects on the accumulation of gluten proteins. The similarity of the responses to the different treatments is likely due to source-sink activities of nitrogen reserves in the wheat plant. Because each protein that showed a response in this study is linked to a gene sequence, the work sets the stage for transgenic studies that will better elucidate the roles of specific proteins in flour quality and in the response to the environment.

## Background

Wheat flour is used worldwide as an ingredient in a variety of baked products due to its unique protein properties. When flour is mixed with water, the proteins interact to form gluten, a continuous protein network that is responsible for the viscoelastic properties and gas holding capacity of flour dough. Gluten is a complex mixture of several hundred proteins with molecular weights ranging from 30,000 to 88,000 Da. The gluten proteins are synthesized in the endosperm of the developing wheat grain and comprise 60–80% of flour protein. Based on extraction and solubility properties, the gluten proteins can be separated into two major groups: the alcohol-soluble gliadins and the alcohol-insoluble glutenins [1]. The gliadins contribute mainly to the viscosity and extensibility of wheat dough and the glutenins to elasticity and dough strength. The monomeric gliadins are classified as α-, γ-, and ω-gliadins based on their mobility in acid PAGE gels. The glutenins are classified as high molecular weight-glutenin subunits (HMW-GS) and low molecular weight-glutenin subunits (LMW-GS), which link together via intermolecular disulfide bonds to form large insoluble polymers. Variations in flour quality are related to genetic differences in gliadin and glutenin composition among wheat cultivars and to effects of environmental conditions on their relative proportions [2,3].

Since consistent flour quality is important to end users, an increased understanding of the effects of environment on individual gliadins and glutenins and their relationship to quality is needed. Due to the difficulties in separating individual gluten proteins by chromatographic methods [4], quantitative two-dimensional gel electrophoresis (2-DE) has become a method of choice for identifying proteins that respond to environmental cues. To date, a number of studies have used 2-DE to discover gluten protein responses to high temperature, fertilizer, or drought [5-11] and have reported changes in as few as three [8] to as many as 107 2-DE spots [11]. Mass spectrometry (MS) was used to identify some of the proteins that showed responses to environmental cues. However, in most studies, identifications were based on very few peptides and it was generally not possible to distinguish individual gluten proteins within the major classes.

Recently, we developed improved methods for tandem mass spectrometry (MS/MS) identification of the gluten proteins. Proteins in 2-DE spots were digested individually with chymotrypsin, thermolysin, and trypsin to increase sequence coverage. In addition, search strategies were improved and databases that included cultivar-specific gluten protein sequences were constructed for analysis of spectral data [12]. This methodology made it possible to develop a comprehensive proteome map of flour from the US wheat Butte 86 in which 233 individual 2-DE spots were assigned to specific gene sequences [13]. Gluten proteins identified included 5 HMW-GS, 22 LMW-GS, 13 γ-gliadins, 23 α-gliadins and 4 ω-gliadins, many of which had sequence coverage greater than 50%. Among the proteins identified in this study were α-, γ- and ω-gliadins that contain extra cysteine residues and may be linked into the glutenin polymer. In addition, α-gliadins containing specific epitopes involved in celiac disease were distinguished from those that do not contain these sequences. The proteome map made possible a detailed analysis of the effects of fertilizer on gliadin and glutenin levels in flour [14]. One-hundred twenty-two gluten protein spots representing 19 gluten protein gene sequences responded significantly to fertilizer: five HMW-GS, six α-gliadins, and four ω-gliadins increased and three LMW-GS and one γ-gliadin decreased.

In this paper, we compare the effects of high temperature and fertilizer singly and in combination on the entire complement of gliadins and glutenins in developing endosperm and in flour from mature grain. The study extends earlier studies in which we analyzed the effects of high temperature and fertilizer on a limited number of gliadins and glutenins in developing endosperm by 2-DE and evaluated flour composition by RP-HPLC [5,6]. Because the Butte 86 proteome map links gluten proteins to gene sequences, the goal of this study was to identify genes encoding proteins that respond to fertilizer and high temperature that can be used in transgenic approaches to determine the roles of specific proteins in flour quality and in the response of the grain to environmental cues.

## Results

### High temperature and fertilizer alter fresh weight and gluten protein levels during grain development

The plant growth regimens used and the comparisons made in this study are summarized in Table 1. All plants were grown at 24/17°C (day/night maximum temperatures) with fertilizer until anthesis, when pots were placed into four groups. Two groups of plants were irrigated with fertilizer, but one group was grown at 24/17°C (MF, moderate temperature with fertilizer regimen) and the other at 37/28°C (HF, high temperature with fertilizer regimen). The remaining two groups of plants were flushed extensively with water to remove fertilizer. Both groups were watered without fertilizer; one group was grown at 24/17°C (MN, moderate temperature without fertilizer regimen) and the other at 37/28°C (HN, high temperature without fertilizer regimen). These growth conditions made it possible to determine the effects of fertilizer (MNvMF), high temperature (MNvHN), and fertilizer plus high temperature (MNvHF) on gluten protein accumulation. It was also possible to evaluate the effects of high temperature on gluten protein accumulation in plants that received fertilizer (MFvHF) as well as the effects of fertilizer under high temperature conditions (HNvHF).

**Table 1:** Key to plant growth regimens and expected results

**Regimen**	**Expected result**
MN^1^vMF^2^	Differences due to fertilizer under moderate temperature.
MNvHN^3^	Differences due to high temperature.
MNvHF^4^	Differences due to high temperature plus fertilizer.
MFvHF	Differences due to high temperature in the presence of fertilizer.
HNvHF	Differences due to fertilizer under high temperature.

^1^ MN, moderate temperature without fertilizer.

^2^ MF, moderate temperature with fertilizer.

^3^ HN, high temperature without fertilizer.

^4^ HF, high temperature with fertilizer.

**Figure 1:**
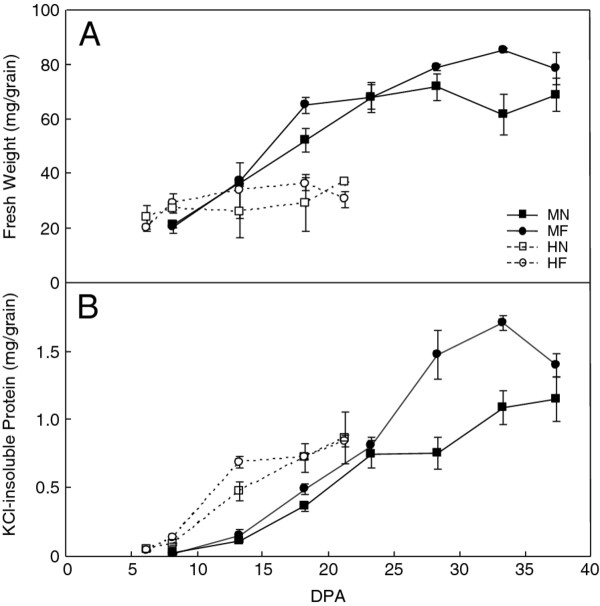
**Effect of fertilizer and high temperature on fresh weight and gluten protein amounts in developing wheat grain. A**. Fresh weight per grain. Grain from two heads per time point were analyzed. **B**. KCl-insoluble gluten protein per grain. Protein was extracted from endosperm collected from each of three heads per time point. MN, MF, HN, and HF are defined in Table 1.

Fresh weight/grain was highest under the 24/17°C regimen with fertilizer (Figure 1A). Grain fresh weight was 79 mg at 37 dpa compared to 69 mg without fertilizer. The 37/28°C regimens shortened the duration of grain fill from 37 to 21 dpa and grain fresh weight was substantially less. Fresh weight/grain at 21 dpa under the high temperature regimens was similar with or without fertilizer. Although mature grain produced under the high temperature regimens was much smaller, the germination rate was 100% (data not shown). Total gluten protein amount/grain, determined by the method of Lowry et al. [15,16] for the KCl-insoluble protein fraction, was highest under the 24/17°C regimen with fertilizer (Figure 1B). At 37 dpa under the 24/17°C regimens, gluten protein amount was somewhat higher with fertilizer than without. At 21 dpa under the 37/28°C regimens, gluten protein amount was similar with or without fertilizer, but substantially less than under the 24/17°C regimens. The high temperature regimens also increased the percentage of gluten protein per grain. With fertilizer, gluten protein was 1.8% of grain fresh weight under the 24/17°C regimen compared to 2.6% under the 37/28°C regimen. Without fertilizer, gluten protein was 1.6% of fresh weight under the 24/17°C regimen compared to 2.2% under the 37/28°C regimen. The increase in the proportion of the gluten proteins per grain under the 37/28°C regimens is due to a decrease in starch levels under the high temperature regimens [17,18].

### Principal component analysis reveals equivalent time points between the 24/17°C and 37/28°C time courses

**Figure 2:**
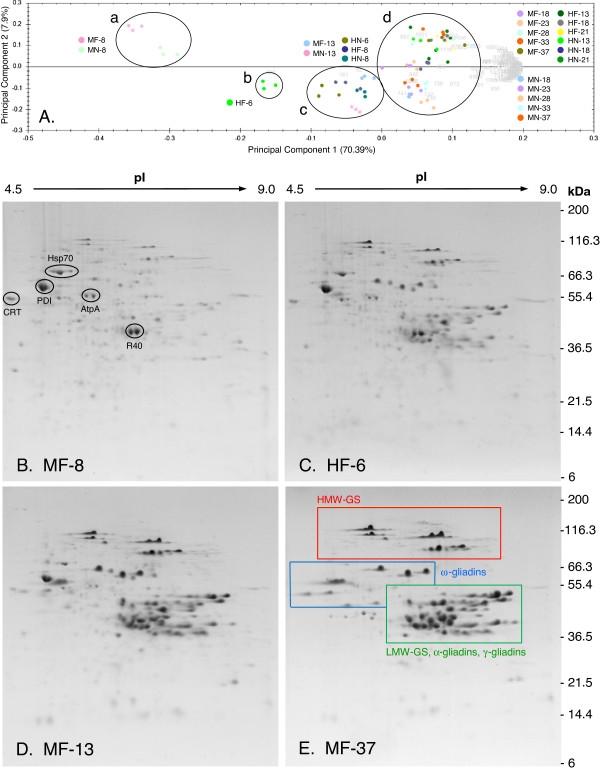
**Two-dimensional gel electrophoresis analysis of gluten proteins during wheat grain development. A**. Principal component analysis. Each symbol corresponds to the gluten protein gel pattern at a single developmental time point. The analysis includes the three replicas for each time point. For cluster d, the keys to the color spots correspond to the location of gel patterns above or below the origin of the y-axis. **B**-**E**. Representative 2-D gels of samples from clusters a, b, c, and d: MF-8, HF-6, MF-13 and MF-37 dpa. MN, MF, HN, and HF are defined in Table 1. Boxes in E outline positions of the HMW-GS, the ω-gliadins, and the LMW-GS plus α- and γ-gliadins. The proteome map and mass spectrometry data for identifications of individual proteins were reported previously [13]. Additional proteins identified in this study are indicated in panel **B**: AtpA, ATP synthase subunit alpha; CRT, calreticulin; Hsp70, 70 kD heat shock-induced protein; PDI, protein disulfide isomerase; R40, ABA- and stress-induced protein (Additional file 2 contains identification data).

Proteins from endosperm collected at seven time points under the 24/17°C regimens and five time points under the 37/28°C regimens were separated by 2-DE. Principal component analysis (PCA) of gluten protein gel patterns from triplicate gels for each time point was used to determine the relationships between the 24 time points comprising the four developmental time courses (Additional file 1 contains representative 2-D gels of all time points for the four treatments). Principal components 1 and 2 accounted for 78.3% of the variation in this analysis (Figure 2A). The three replicate gels for each time point clustered together, demonstrating excellent technical reproducibility of the 2-D gel patterns. PCA separated the gel patterns into four clusters that were aligned from the earliest to the latest time points along the x-axis. Cluster a contained the 8 dpa gel patterns for the 24/17°C regimens with or without fertilizer. Cluster b contained the 6 dpa gel patterns for the 37/28°C regimen with fertilizer. Clusters c and d contained the gel patterns for the remaining time points. Within cluster c, the gel patterns for the 24/17°C regimens with or without fertilizer at 13 dpa were related to those for the 37/28°C regimens at 8 dpa with fertilizer and 6 and 8 dpa without fertilizer. Within cluster d, the gel patterns for the 24/17°C regimen without fertilizer at 18, 23, 28, 33 and 37 dpa were located below the origin of the y-axis and the gel patterns for the 24/17°C regimen with fertilizer at 18, 23, 28, 33 and 37 dpa and the 37/28°C regimens with and without fertilizer at 13, 18 and 21 dpa were located above the origin. This distribution suggests that the 24/17°C regimen without fertilizer was different from the other regimens and that it was a suitable treatment for determining gluten protein responses to fertilizer and high temperature.

Comparison of the 2-D gel patterns of proteins in the developing endosperm provided a partial explanation for the temporal distribution of the clusters generated by PCA (Figures 2B-E). Under the 24/17°C regimen at 8 dpa, the most abundant proteins were ATP synthase A (AtpA), calreticulin (CRT), 70 kD heat shock protein (Hsp70), protein disulfide isomerase (PDI), and stress responsive protein R40 (R40) (Figure 2B). PDI and Hsp70 were identified previously [13,19] and ATP synthase A (AtpA), calreticulin (CRT), and stress responsive protein R40 (R40) were identified in this study (Additional file 2 contains the MS/MS identification data). In keeping with the onset of gluten protein biosynthesis, CRT, Hsp70, and PDI, which reside in the ER, facilitate the folding and maturation of proteins. CRT also modulates calcium homeostasis and Hsp70 assists in transport of precursor proteins into organelles and targets damaged proteins for degradation [20]. PDI regulates the formation, reduction, and isomerization of disulfide bonds associated with protein folding and AtpA is a key enzyme in bioenergetics. The specific role of the R40 proteins remains unclear. It has been proposed that these ABA- and osmotic stress-responsive proteins [21,22] have both protein- and carbohydrate-binding domains enabling them to cross-link proteins and glycoconjugates [23]. The non-gluten proteins appear to decrease throughout development, but this is due to a concomitant large increase in gluten protein accumulation that masks the presence of most non-gluten proteins. In an earlier study [19], a fraction in which gluten proteins were depleted was essential for analysis of non-gluten protein accumulation during Butte 86 grain development. Under the 24/17°C regimens, gluten proteins were present at very low levels at 8 dpa (Figure 2B), but comprised a major proportion of proteins from 13 to 37 dpa (Figure 2D and E, Additional file 1). Under the 37/28°C regimens, gluten protein levels were more abundant at 6 dpa (Figure 2C) than at 8 dpa under the 24/17°C regimens and comprised a major proportion of proteins from 8 to 21 dpa (Additional file 1).

### High temperature and fertilizer alter individual gliadin and glutenin accumulation profiles during grain development

**Figure 3:**
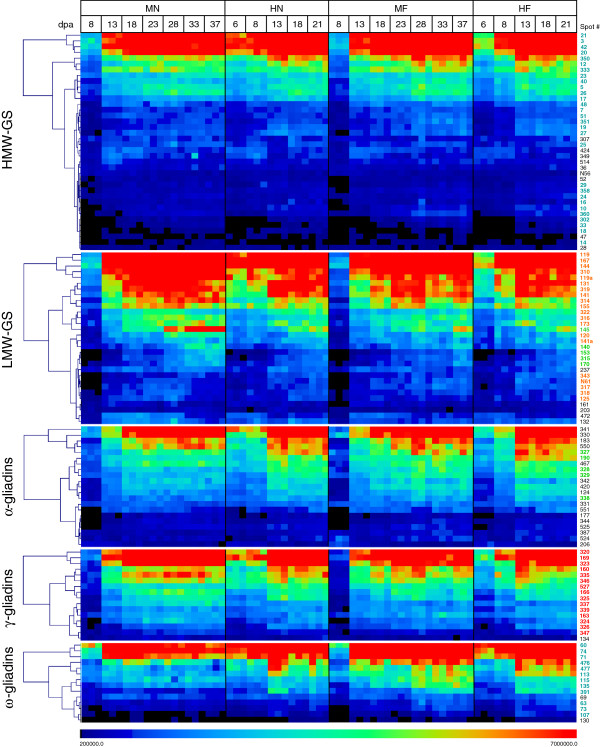
**Accumulation patterns of gluten proteins during wheat endosperm development.** Accumulation profiles were analyzed by hierarchical cluster analysis and visualized using the TIGR Multiexperiment Viewer. Each square represents the normalized volume of a single 2-D gel spot. The color scale indicates relative protein amount. The dendrograms show the relationships between the profiles within each of the gluten protein classes. Additional file 3 contains the data set of normalized spot volumes. Spot numbers are color coded to match the subset of proteins selected by correlation analysis shown in Figure 4. HMW-GS N56 and the LMW-GS N61 were identified in developing endosperm and not detected in flour; Additional file 1, Figure MN-13 shows the map location and Additional file 2 contains the mass spectrometry data for these proteins. MN, MF, HN, and HF are defined in Table 1.

Using protein identifications of 2-DE spots determined by MS/MS in Dupont et al. [13], protein profiles were grouped into the HMW-GS, LMW-GS, and the α-, γ-, and ω-gliadins. These were examined by hierarchical cluster analysis to provide a comprehensive image of the accumulation patterns of the gluten proteins during endosperm development under the four environmental regimens (Figure 3; original data in Additional file 3). The resulting display contains 8,208 data points: 24 experimental time points, 114 gluten protein spots, and spot volumes from triplicate 2-D gels. The spot volumes for the replicates clustered together at each time point, demonstrating excellent technical reproducibility of the 2-D gel patterns. The hierarchical cluster analysis ordered the accumulation profiles for each protein class by protein amount, which had a dynamic range of six orders of magnitude. Due to this wide dynamic range, a color scale of 200,000 to 7,000,000 was chosen to display the spot volume data. Depending on gluten protein class, 0.6-5.4% of the protein profiles with spot volumes less than 200,000 are off-scale and 7.7-16% with spot volumes greater 7,000,000 are off-scale. The dendrograms in Figure 3 show the relationships between the protein profiles within each of the gluten protein classes. The identities of the gluten proteins corresponding to the profiles depicted in Figure 3 are located in Table 2 and are listed in Additional file 3 in the order determined by the hierarchical cluster analysis. Profiles of gluten protein accumulation under the 24/17°C regimens appeared similar with or without fertilizer. Most HMW-GS, LMW-GS, and α-, γ-, and ω-gliadins increased rapidly between 8 and 13 dpa and then increased gradually from 13 to 37 dpa. The 37/28°C regimens compressed the developmental profiles; proteins increased rapidly between 6 and 8 dpa and attained maximal levels from 13 to 21 dpa.

**Table 2:** Effect of temperature and fertilizer on accumulation of individual gluten proteins in flour

**Predominant protein**^**1**^	**# Spots**	**Spot numbers**^**5**^	**Spot volume × 10**^**–5 (6)**^								**% Change**^**11**^				
			**MN**^**7**^		**MF**^**8**^		**HN**^**9**^		**HF**^**10**^		**MNvMF**	**HNvHF**	**MNvHN**	**MNvHF**	**MFvHF**
**HMW-GS**			**Ave**	**SD**	**Ave**	**SD**	**Ave**	**SD**	**Ave**	**SD**					
Ax2†	8	12, 16, 17, 18, 19, 23, 24, 28	66.8	2.8	91.3	2.7	91.0	1.2	94.5	6.3	**36.7**	3.8	**36.3**	**41.6**	3.5
Bx7†	8	20, 21, 25, 26, 27, 29, 33, 302	136.3	6.7	170.6	2.7	164.8	2.0	161.6	8.1	**25.2**	−2.0	**21.0**	18.6	−5.3
Dx5†	8	3, 5, 7, 10, 14, 36, 307, 538	96.7	0.9	121.6	5.3	128.9	0.2	130.1	5.9	**25.7**	0.9	**33.4**	**34.6**	7.0
By9†	8	40, 47, 52, 349, 350, 351, 358, 360	50.7	1.8	75.6	3.5	77.8	2.4	77.0	5.6	**49.2**	−1.1	**53.6**	**52.0**	1.8
Dy10†	6	42, 48, 51, 333, 424, 514	88.3	6.0	104.7	8.4	105.2	6.0	98.1	10.5	18.6	−6.7	19.1	11.1	−6.3
**LMW-GS**^**2**^															
Bu-1 (m-type)	2	167, 170‡	94.9	4.6	99.2	6.2	94.5	4.2	98.1	7.1	4.5	3.8	−0.5	3.3	−1.1
Bu-3† (s-type)	9	119, 119a, 120, 131, 132, 161, 237, 310, 316	282.5	7.5	258.1	7.5	247.1	13.8	237.3	4.8	−8.6	−3.9	−12.5	−16.0	−8.1
Bu-4 (i-type)	3	140‡, 141, 141a	76.8	3.1	82.9	0.5	82.2	7.1	83.0	2.8	8.0	0.9	7.1	8.0	0.0
Bu-6† (m-type)	1	173	35.1	4.1	27.0	2.8	30.5	6.0	26.9	2.4	***−23.1***	−11.6	−13.1	***−23.2***	−0.1
Bu-7† (m-type)	3	144, 145‡, 472	102.6	4.6	88.3	5.5	93.5	8.9	87.7	3.2	−14.0	−6.1	−8.9	−14.5	−0.7
Bu-8 (m-type)	1	343	3.4	0.3	2.8	0.1	3.1	0.8	3.3	0.7	−18.7	6.6	−10.1	−4.2	17.9
Bu-11 (m-type)	2	203, 315‡	17.7	1.1	12.2	1.5	11.0	1.3	12.3	1.3	***−31.4***	11.6	***−37.9***	***−30.7***	1.1
Bu-18 (m-type)	2	153‡, 155	40.1	4.3	35.4	3.3	37.1	6.9	33.6	1.9	−11.7	−9.6	−7.4	−16.2	−5.1
Bu-2/13 (s-type)	4	314, 317, 318, 322	92.8	7.7	101.1	3.3	98.2	3.2	90.6	4.9	9.0	−7.7	5.8	−2.3	−10.4
GenBank: AAB48469 (i-type)	1	125	6.5	0.2	6.1	0.4	7.4	0.2	7.7	1.0	−6.3	3.9	14.5	18.9	**26.8**
TC11-277270 (m-type)	1	319	31.6	3.6	29.8	2.7	35.6	4.2	32.5	1.2	−5.7	−8.7	12.5	2.8	9.0
**α-Gliadins**															
Bu-1†	1	342	11.5	0.6	14.7	1.8	12.3	10.6	11.2	1.0	**27.9**	−9.4	7.6	−2.5	***−23.7***
Bu-2^3^	2	330, 338	86.8	1.1	97.8	2.9	87.0	1.5	90.4	4.0	12.6	3.9	0.2	4.1	−7.5
Bu-3†	1	468	40.4	1.5	46.7	5.6	42.9	0.5	43.5	3.0	15.6	1.5	6.0	7.6	−6.9
Bu-4†	1	467	53.6	3.1	62.0	2.5	59.9	1.3	58.4	2.7	15.7	−2.5	11.7	8.9	−5.9
Bu-5	2	183, 344	38.6	0.3	46.9	2.0	48.3	3.0	46.5	5.2	**21.7**	−3.7	**25.2**	**20.5**	−1.0
Bu-10	1	550	27.9	1.4	33.8	1.3	31.5	8.0	32.6	3.0	**21.5**	3.6	13.0	17.1	−3.6
Bu-11	1	327	47.4	4.0	69.6	4.4	67.0	7.6	69.0	4.7	**46.7**	3.0	**41.3**	**45.5**	−0.8
Bu-12†^4^	5	328, 329, 387, 524, 525	78.6	2.3	118.1	1.9	106.7	10.2	114.7	3.9	**50.2**	7.5	**35.7**	**45.9**	−2.9
Bu-14†	4	190, 206, 331, 551	33.3	0.5	42.5	2.4	45.2	1.6	48.3	6.8	**27.5**	7.0	**35.7**	**45.2**	13.8
Bu-23^4^	1	341	92.0	3.3	97.1	2.4	95.0	5.2	91.6	3.9	5.5	−3.6	3.2	−0.5	−5.7
Bu-27	1	420	14.7	1.4	17.8	2.1	17.5	1.5	16.3	0.9	**21.2**	−7.2	19.1	10.5	−8.9
Bu (GenBank:BQ806209)	1	177	7.0	0.5	5.9	1.3	5.1	0.7	4.5	0.5	−16.0	−12.2	***−27.1***	***−36.0***	***−23.7***
Bu (GenBank:BQ807130)†	1	124	11.4	1.3	15.3	2.6	16.1	1.7	15.3	0.4	**33.9**	−5	**41.1**	**34.1**	0.1
**γ-Gliadins**															
Bu-1†	1	326	4.9	0.4	3.5	0.7	3.9	1.7	3.1	0.6	***−28.1***	−19.8	***−20.5***	***−36.2***	−11.3
Bu-2	1	160	72.4	2.2	80.7	3.0	84.4	3.5	87.9	3.5	11.4	4.2	16.5	**21.4**	9.0
Bu-4^3^	3	166, 169, 337	92.4	5.2	85.6	8.0	92.7	8.9	84.8	0.4	−7.4	−8.5	0.2	−8.3	−0.9
Bu-5	4	134, 320, 323, 324	142.9	5.1	132.7	6.6	132.9	9.8	127.9	6.9	−7.1	−3.8	−7.0	−10.5	−3.6
Bu-6	3	335, 346, 347	67.4	8.7	72.2	2.4	78.3	6.1	76.2	4.0	7.1	−2.7	16.3	13.1	5.5
Bu-7	1	339	10.6	1.4	9.6	1.1	9.5	0.8	7.8	1.2	−9.7	−17.6	−10.6	***−26.4***	−18.5
Bu-11	2	163, 527	32.3	1.1	32.2	1.6	36.5	3.3	37.0	0.9	−0.2	1.4	13.1	14.7	15.0
Bu-1 or Bu-8^3^	1	325	31.3	1.5	34.6	1.8	32.9	5.0	32.0	3.8	10.5	−2.8	5.4	2.5	−7.3
**ω-Gliadins**															
Gli-B3 type (ω-5)†	6	60, 63, 69, 71, 73, 74	110.9	2.8	230.3	2.6	180.9	20.9	186.1	20.9	**107.6**	2.9	**63.1**	**67.8**	−19.2
Gli-D3 type (ω-1, 2)†	2	476, 477	60.1	3.4	109.4	5.3	108.2	15.5	106.7	15.5	**82.0**	−1.4	**80.1**	**77.5**	−2.4
Bu-D5 (ω-1, 2)†	2	135, 391	25.5	2.5	50.8	4.4	45.7	10.1	49.5	10.1	**99.2**	8.2	**79.5**	**94.3**	−2.5
Cys type TC-262770 (ω-1, 2)†^3^	4	107, 113, 115, 116	41.2	3.7	64.2	5.1	63.6	9.5	66.6	9.5	**56.0**	4.6	**54.6**	**61.7**	3.6
secalin-like	1	130	3.5	0.5	3.6	0.5	3.1	0.4	3.5	0.4	2.7	13.0	−10.1	1.6	−1.0

^1^ Proteins were identified in Dupont et al. [13].

^2^ LMW-GS are classified as m-, s- or i-type based on their N-terminal amino acid sequence.

^3^ Denotes gliadins containing odd numbers of cysteines.

^4^ Denotes α-gliadins that do not contain Glia-α, Glia-α2, Glia-α-9 or Glia-α-20 celiac disease epitopes.

^5^ Spots containing same gene sequences.

^6^ Ave and SD refer to average and standard deviation of normalized volumes from all spots with the same identification. Additional file 4 contains the data set of normalized spot volumes.

^7^ MN, 24/17°C minus fertilizer.

^8^ MF, 24/17°C plus fertilizer.

^9^ HN, 37/28°C minus fertilizer.

^10^ HF, 37/28°C plus fertilizer.

^11^Increases of 20% or more are indicated in bold type and decreases of 20% or more are indicated in bold italics type.

† Proteins that responded significantly to fertilizer in Altenbach et al. [14].

‡ Spot numbers of proteins comprising LMW-GS_B_, a group of LMW-GS sharing a unique developmental profile.

**Figure 4:**
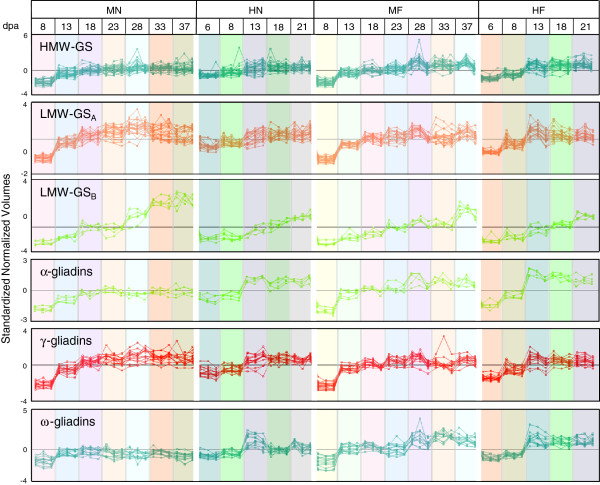
**Correlation analysis of accumulation profiles of gluten proteins in developing wheat endosperm.** The profile colors correspond to the spot numbers in Figure 3 and may be used with Table 2 and Additional file 3 to determine the identities of the individual proteins within the gluten protein classes. MN, MF, HN, and HF are defined in Table 1.

Because individual profiles were difficult to distinguish in Figure 3, correlation analysis was used to investigate accumulation profiles in more detail. Protein profiles were grouped into HMW-GS, LMW-GS, and the α-, γ-, and ω-gliadins, spot volumes log normalized, and accumulation patterns analyzed (Figure 4). The profiles include the three replicate spot volumes for each time point and are color coded to match the spot numbers in Figure 3. This analysis showed, with the exception of a few outliers, that the profiles for the HMW-GS and the γ- and ω-gliadins formed single clusters under each of the environmental treatments while the LMW-GS formed two clusters. The α-gliadins exhibited greater variability and only five of the 21 profiles clustered together.

Under the 24/17°C regimen without fertilizer, the gluten proteins exhibited four developmental profiles (Figure 4, MN). The HMW-GS and α-gliadins reached maximum levels at 18 dpa and remained at these levels until 37 dpa. The ω-gliadins attained maximum levels at 13–18 dpa and declined slightly by 37 dpa. The majority of LMW-GS (Figure 4, LMW-GS_A_) had profiles similar to the γ-gliadins and attained maximum levels at 28 dpa and declined slightly by 37 dpa. A small group of LMW-GS (spots 140, 145, 153, 170, 315) increased gradually throughout development and attained maximum levels at 37 dpa (Figure 4, LMW-GS_B_, indicated by ‡ in the Spot Numbers column in Table 2). These spots were identified as the m-type LMW-GS Bu-1, Bu-7, Bu-11 and Bu-18 and the i-type LMW-GS Bu-4 [13]. Under the 24/17°C regimen with fertilizer, the gluten proteins exhibited only two developmental profiles (Figure 4, MF). The HMW-GS, LMW-GS_A_, and the α-, γ-, and ω-gliadins peaked at 28 and the LMW-GS_B_ were maximal at 37 dpa. Compared to the 24/17°C regimen without fertilizer, the HMW-GS, α-gliadins, and ω-gliadins were higher from 28–37 dpa, the LMW-GS_A_ were lower from 28–37 dpa, the LMW-GS_B_ were lower from 18–37 dpa, and the γ-gliadins were somewhat lower at 33 dpa (Figure 4, compare MN and MF). At 37 dpa, the most notable changes in gluten protein levels were increases in the α- and ω-gliadins and decreases in the LMW-GS_B_.

Under the 37/28°C regimens, the gluten proteins exhibited three developmental profiles without and with post-anthesis fertilizer (Figure 4, HN and HF). The HMW-GS and LMW-GS_A_ attained maximum levels at 13 dpa and remained at these levels until 21 dpa. The α-, γ-, and ω-gliadins also peaked at 13 dpa, but declined slightly by 21 dpa. The LMW-GS_B_ increased gradually throughout development and attained maximum levels at 21 dpa. The most notable changes in gluten protein levels at 21 dpa under the two high temperature regimens were increases in the ω-gliadins.

### High temperature and fertilizer alter levels of specific gliadins and glutenins in flour

Since flour quality depends on gluten protein composition, the effect of fertilizer and high temperature on gluten protein levels was examined in flour milled from mature grain harvested from plants used for the developmental studies (original data in Additional file 4). Because the same protein sequences were often associated with multiple 2-D spots, the volumes of spots containing proteins with the same identifications were summed for this analysis as was done by Altenbach et al. [14]. This resulted in 42 different gluten proteins (Table 2). The percent change in volume relative to the 24/17°C regimen without fertilizer was calculated to determine specific gluten protein responses to fertilizer, high temperature, and high temperature plus fertilizer. In a previous study, 19 gluten proteins showed statistically significant changes of 20% or more in response to fertilizer under a 24/17°C regimen (Table 2, indicated by † in the Predominant Protein column) [14]. In this study, it is notable that 14 proteins responded not only to fertilizer (MNvMF), but also to high temperature (MNvHN) and high temperature plus fertilizer (MNvHF). These included three HMW-GS (Ax2*, Dx5, By9), one m-type LMW-GS (Bu-11), five α-gliadins (Bu-5, Bu-11, Bu-12, Bu-14, Bu-BQ807130), one γ-gliadin (Bu-1) and one ω-5 gliadin and three ω-1,2 gliadins.

**Figure 5:**
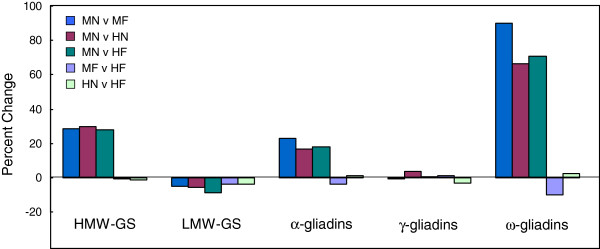
**Effect of temperature and fertilizer on proportion of gluten protein classes in flour.** Comparisons of percent change in relative volumes of gluten protein classes were made for MNvMF, MNvHN, MNvHF, MFvHF and HNvHF. Additional file 4 contains the data set of normalized spot volumes. MN, MF, HN, and HF are defined in Table 1.

The effects of fertilizer and high temperature were not additive. In fact, only three low abundance proteins differed 20% or more in amount when plants that received fertilizer were subjected to high temperature (MFvHF); an i-type LMW-GS (GenBank: AAB48469) increased and two α-gliadins (Bu-1, Bu-BQ806209) decreased. In addition, protein abundance changed little when plants subjected to high temperature received fertilizer (HNvHF). A comparison of percent change of the gluten protein groups (Figure 5) in response to fertilizer, temperature, and high temperature plus fertilizer showed that the HMW-GS, α-gliadins, and ω-gliadins increased, the LMW-GS decreased, and the γ-gliadins showed little change in response to fertilizer, high temperature or fertilizer plus high temperature. The increases were greatest for the ω-gliadins and much less for the HMW-GS and α-gliadins. After accounting for gliadins containing extra cysteines (indicated in Table 2), ratios of gliadins to glutenins increased from 0.71 for the MN treatment to 0.86, 0.81 and 0.84 for the MF, HN and HF treatments, respectively. Ratios of HMW-GS to LMW-GS also increased from 0.44 for the MN treatment to 0.57, 0.58 and 0.59 for the MF, HN and HF treatments, respectively.

## Discussion

### Effect of fertilizer and high temperature on developmental profiles of gluten proteins

Quantitative 2-DE was used to determine the effects of post-anthesis fertilizer and high temperature on the accumulation of individual gliadins and glutenins in the developing endosperm and in flour milled from the mature grain. To distinguish responses to temperature from those of fertilizer, plants were grown under 24/17°C and 37/28°C regimens without and with post-anthesis fertilizer. 2-DE spots identified as HMW-GS, ω-gliadins or γ-gliadins generally showed expression profiles characteristic of each protein group while spots identified as α-gliadins showed a variety of expression profiles. Individual LMW-GS spots exhibited two distinctly different developmental profiles. One group (LMW-GS_A_) had expression profiles similar to the γ-gliadins and another group (LMW-GS_B_) increased gradually throughout development. Of the five proteins comprising LMW-GS_B_, four were identified by MS/MS as m-type LMW-GS (Bu-1, Bu-7, Bu-11, Bu-18) and one as an i-type LMW-GS (Bu-4) (Table 2). These proteins had the same MS/MS identities as other proteins in the LMW-GS_A_ group, but had different pIs and M_r_s. Although MS/MS sequence coverage for these LMW-GS was quite high, generally about 50%, differences in the expression profiles suggest that very similar proteins in the LMW-GS_A_ and LMW-GS_B_ groups are encoded by different genes. Juhász et al. [24] recently found two expression profiles for LMW-GS genes. However, they reported that s-type and i-type LMW-GS had expression profiles similar to the LMW-GS_B_ in the present study. Liu et al. [25] also reported multiple expression profiles for LMW-GS genes in a proteomic analysis of three Chinese wheat cultivars.

The developmental profiles demonstrate that gluten protein accumulation is a complex process that is subject to spatial and temporal regulation as well as environmental signaling. Individual proteins within each gluten protein class accumulated to different levels, suggesting that the corresponding genes had different basal levels of expression. Ultimately, the proportions of ω-gliadins and HMW-GS and a few α-gliadins and LMW-GS changed in flour in response to both post-anthesis fertilizer and high temperature while those of most α- and γ-gliadins and LMW-GS were unchanged. The data suggest that there are differences in the promoter sequences of the gluten protein genes, both between and within the major gene families. Promoter elements responsible for tissue specificity of the gluten proteins are the endosperm box, which is comprised of the prolamin box and a GCN4-like motif (GLM), and the AACA motif [26,27]. GLM, also known as the N motif, is thought to play a role in the response of the gluten protein genes to nitrogen [28,29]. Recently, Juhász et al. [24] compared regulatory elements in the 5^′^ non-coding regions of LMW-GS genes available in public databases and developed a model for their transcriptional regulation based on an *in silico* analysis of expression profiles. Clearly, it will be important to confirm this model using experimental data and extend the model to include other classes of gluten protein genes. Comparisons of promoter regions from genes encoding gluten proteins characterized in this study should prove valuable for this type of analysis.

### Effect of fertilizer and high temperature on gluten protein composition in flour

Dupont et al. [6] used RP-HPLC to quantify the effects of fertilizer and high temperature on the accumulation of Butte 86 flour proteins and reported changes in the levels of ω-gliadins and HMW-GS. However, changes in specific LMW-GS, α- and γ-gliadins were difficult to detect because of the complexity of these groups of proteins. In this study, 2-DE analysis revealed a number of changes in distinct gluten proteins within these classes in response to fertilizer and temperature. Of the 42 uniquely identified gluten proteins, 16 increased with fertilizer under moderate temperatures (four HMW-GS, eight α-gliadins, and four ω-gliadins) and three decreased (two LMW-GS and one γ-gliadin). The response to the 37/28°C regimen without fertilizer (MNvHN) was comparable to that of 24/17°C regimen with fertilizer (MNvMF); thirteen of the same proteins increased (four HMW-GS, five α-gliadins, and four ω-gliadins) and two of the same proteins decreased (one LMW-GS and one γ-gliadin). Most of these proteins also responded to high temperature plus fertilizer (MNvHF). It is notable that the effects of fertilizer and high temperature were not additive and that very few significant changes were observed when plants receiving fertilizer were subjected to high temperature (MFvHF). Likewise, the application of fertilizer under a high temperature regimen had little effect on gluten protein accumulation (HNvHF). As noted previously [5,14,30,31], the response to high temperature and fertilizer is related to the proportion of the S-containing amino acids in the different gluten protein classes. Generally, the proportions of low S-proteins (ω-gliadins) and low to medium S-proteins (HMW-GS and α-gliadins) increased and those of the high S-proteins (LMW-GS and γ-gliadins) remained the same or decreased.

In agreement with previous studies [6,14,32], the ratios of gliadins to glutenins increased in response to fertilizer and high temperature. The higher ratios reflect increases in the proportions of ω-gliadins and certain α-gliadins. The ratios of the HMW-GS to LMW-GS also increased in response to both fertilizer and high temperature, largely due to increases in HMW-GS and decreases in certain LMW-GS. Of the gliadins containing an odd number of cysteines and likely to function as LMW-GS, only the cys-type ω-gliadins showed responses to fertilizer or high temperature. Others have reported that the ratio of the HMW-GS to LMW-GS in a glutenin polymer fraction of SDS unextractable protein is significantly correlated with dough strength [33-37].

Because individual proteins in this analysis were associated with specific gene sequences, changes in the immunogenic potential of the flour that result from the growth conditions of the plant can now be assessed. It is notable that the *Gli-B3* ω-gliadins (also referred to as ω-5 gliadins) are associated with the food allergy wheat-dependent exercise-induced anaphylaxis. These proteins increased significantly in response to both fertilizer and temperature. Among the gluten proteins, the α-gliadins are considered most immunogenic in celiac disease. Several of the α-gliadins that increased in response to both fertilizer and temperature contain epitopes important in celiac disease (Bu-5, Bu-11, Bu-14) while others do not contain these sequences (Bu-12) (Table 2) [13,38]. It is interesting that proteins that do not contain celiac epitopes comprised 31–32% of the total α-gliadins under all treatments, suggesting that neither fertilizer nor temperature influenced the immunogenic potential of the flour with respect to celiac disease. The accumulation of other immunogenic proteins were not assessed in this study. However, Altenbach et al. [14] analyzed a fraction of total SDS-extractable protein and reported changes in the proportions of other proteins with fertilizer that are likely food allergens. These included increases in serpins and decreases in lipid transfer protein (LTP), chitinase, the α-amylase/trypsin inhibitor CM3 and globulin-2. Hurkman et al. [19] analyzed an albumin/globulin fraction and reported that the same proteins had opposite responses to high temperature when plants were supplied with fertilizer. They observed increases in LTP, chitinase and globulin-2 and decreases in the proportions of several serpins.

### Source-sink activities and gluten protein responses to fertilizer and high temperature

The question arises as to why post-anthesis high temperature and the post-anthesis addition of fertilizer alter gluten protein composition in the same manner. The similarity in responses of the gliadins and glutenins to high temperature and fertilizer might be explained by source-sink activities in the wheat plant [39-45]. All plants were grown with fertilizer and adequate water under moderate temperatures until anthesis so the amount of nitrogen and carbon reserves that could be mobilized to the developing grain for the synthesis of protein and starch were comparable at the beginning of the treatments. The addition of post-anthesis fertilizer enhances the remobilization of reserves during grain fill and the rate of protein accumulation in the grain, resulting in increases in flour protein percentage and specific changes in gliadin and glutenin composition in flour [5,6,14]. On the other hand, high temperature conditions elicit premature leaf senescence and shorten the period of grain fill [39]. Because remobilization of nitrogen reserves to the developing grain occurs over a significantly shorter period of time, the temperature treatment may essentially mimic high nitrogen conditions, resulting in changes in the same complement of gluten proteins. The lack of response to fertilizer under high temperature conditions suggests that the plants are unable to utilize fertilizer under the temperatures used in this study. This would also explain why post-anthesis fertilizer and temperature do not elicit an additive response.

## Conclusions

This study provides insight into the manner in which specific growth conditions during wheat grain development influence the accumulation of the individual gluten proteins that determine flour quality. While many gluten proteins respond to fertilizer and high temperature, most of the changes are small in magnitude and surprisingly similar between the two treatments. It is notable that both fertilizer and high temperature increase the proportions of most HMW-GS and ω-gliadins and some of the same α-gliadins while decreasing the proportions of several LMW-GS and a minor γ-gliadin. A critical question is how the observed changes in gluten protein composition influence flour quality. Many different proteins contribute to flour quality. While the roles of HMW-GS have received considerable attention, the functions of individual LMW-GS and gliadins in flour quality remain poorly defined. The identification of genes within these classes that respond to fertilizer or temperature now makes it possible to utilize transgenic approaches to determine the importance of specific proteins in flour quality and in the response of the grain to the environment. For example, RNA interference was used recently to create transgenic Butte 86 plants in which genes encoding the ω-5 gliadins were silenced [46]. Since these proteins show some of the largest responses to fertilizer and temperature, analyses of transgenic lines should elucidate how the proteome responds to fertilizer and high temperature in their absence and reveal the contribution of the ω-5 gliadins to flour quality.

## Methods

### Plant material

*Triticum aestivum* L. cv. Butte 86 was grown in climate-controlled greenhouses as described previously [14,47]. Seven seeds were sown per pot (25 cm diameter by 24 cm high). The pots contained Sunshine Mix Number 1 (SunGro Horticulture, Inc., Bellevue, WA, USA), a planting mix that contains 70–80% Sphagnum peat moss plus perlite, dolomitic limestone, gypsum, and wetting agent. Prior to anthesis, plants were grown at a maximum daytime temperature of 24°C and a minimum nighttime temperature of 17°C. Pots were watered by drip irrigation with 20-20-20 fertilizer (Plantex, Plant Products Co. Ltd., Brampton, Ontario, Canada). Pots received 500 mls of 0.6 g/l fertilizer or 0.06 nitrogen units per day. Pots did not receive S. S contents of flour from grain produced under the same environmental conditions indicated that S was not limiting [5]. At anthesis, pots were divided into four groups. One group of pots was left in the 24/17°C greenhouse and irrigated with fertilizer. The second group of pots was flushed with water to remove fertilizer, returned to the 24/17°C greenhouse, and hand-watered at the same rate without fertilizer. The third group of pots was transferred to a second greenhouse that had a maximum daytime temperature of 37°C and a minimum nighttime temperature of 28°C and supplied with fertilizer. The fourth group of pots was flushed with water, transferred to the 37/28°C greenhouse, and hand-watered without fertilizer. Wheat heads were harvested throughout grain fill. Grain number per head ranged from 29–49. For the 24/17°C regimens, heads were collected at 4 to 5-day intervals from 8 to 37 dpa. Because high temperature accelerated the grain developmental program, heads were collected at 2 to 5-day intervals from 6 to 21 dpa for the 37/28°C regimens. The region of the grain containing the embryo was first excised with a razor blade. The endosperm was squeezed through the resultant opening onto a spatula, leaving most of the pericarp/testa/aleurone behind. Endosperm was transferred immediately into tubes cooled in liquid nitrogen and stored at −80°C [19,48]. For developmental studies, the end point for each regimen was the oldest age that endosperm could be squeezed or scraped from the grain after the embryo was excised. Flour was milled from mature grain samples (100 g) with a Brabender Quadrumat Junior (South Hakensack, NJ) at the Hard Winter Wheat Quality Laboratory (US Department of Agriculture, Agricultural Research Service, Manhattan, KS). Flour protein content was determined by NIR and was 7.7 for MN, 14.0 for MF, 15.9 for HN, and 17.7 for HF [6]. Flour was stored in sealed containers at −80°C.

### Protein preparation

A KCl-insoluble fraction enriched in gliadins and glutenin subunits was prepared from endosperm collected at each time point as described previously [48]. Fifty mg of endosperm was ground to a fine powder in liquid nitrogen using a mortar and pestle and suspended in 200 μl of cold (4°C) KCl buffer containing protease inhibitors (1 tablet per 10 ml of Mini Complete Protease Inhibitor Cocktail, Roche Applied Science, Indianapolis, IN). The suspensions were incubated on ice for 5 min with intermittent mixing and centrifuged at 14,000 g for 15 min at 4°C. The pellet or KCl-insoluble fraction was suspended in 800 μl of SDS buffer (2% SDS, 10% glycerol, 50 mM DTT, 40 mM Tris-Cl, pH 6.8), incubated for 1 h at room temperature, and insoluble material removed by centrifugation at 14,000 g for 10 min at room temperature (Eppendorf 5415C; Brinkman Instruments, Inc., Westbury, NY). Proteins were then precipitated by addition of 4 vol of cold (−20°C) acetone and incubation overnight at −20°C to remove SDS, which interferes with protein determination and prevents separation of proteins by IEF. Samples were centrifuged at 14,000 g for 10 min at room temperature, the pellet was rinsed by pipetting cold acetone onto the pellet, centrifuging at 14,000 rpm for 10 min at room temperature, and pipetting the acetone off of the pellet. The pellet was dried at room temperature and solubilized in urea buffer (9 M urea, 4% NP-40, 1% DTT, and 2% ampholytes) to a final concentration of 3 μg protein/μl. For protein determinations, triplicate 5 μl samples were removed from the SDS extracts, precipitated with acetone, and, following addition of 0.1 N NaOH, protein amount assayed by the procedure of Lowry et al. [16] as described by Hurkman and Tanaka [49].

Flour was extracted with SDS buffer (2% SDS, 10% glycerol, 50 mM DTT, 40 mM Tris-Cl, pH 6.8) as described in detail by Dupont et al. [13]. Protein amounts from 50 mg of flour from MN, MF, HN and HF treatments were determined using the Lowry assay [16] and were 3.29, 5.38, 6.14 and 6.64 mg, respectively.

### Separation of proteins by 2-DE

Endosperm and flour proteins were separated by 2-DE as described previously [15,49]. Briefly, samples were suspended in urea buffer (9 M urea, 4% NP-40, 1% DTT, and 2% ampholytes) at a concentration of 3 μg protein/μl. The first dimension capillary tube gels contained 9.2 M urea, 4% (total monomer) acrylamide:BIS, 2% Nonidet P-40, 2% 3–10 Iso-Dalt Grade Servalyts (Crescent Chemical Co., Islandia, NY), 0.015% ammonium persulfate, and 0.125% TEMED. Triplicate IEF gels were loaded with 18 μg of protein and run using a Mini Protean II Tube Cell (BioRad Laboratories, Richmond, CA). Gels were extruded into microcentrifuge tubes and equilibration buffer (2.3% SDS, 10% glycerol, 0.05% dithiothreitol, and 62.5 mM Tris-Cl, pH 6.8) added. The gels were frozen by placing the tubes in crushed dry ice and then stored at −70°C until use. Proteins were separated in the second dimension by SDS gel electrophoresis using an XCell SureLock Mini-Cell electrophoresis system with Novex NuPage 4–12% acrylamide Bis-Tris gels and NuPAGE MES SDS running buffer (Invitrogen Corp., Carlsbad, CA). Gels were stained with Coomassie G-250 (Sigma, St. Louis, MO), destained in water for 2 h at room temperature and stored at 4°C in 20% ammonium sulfate.

### Statistical analysis of 2-D gel images

2-DE gels were digitized with a calibrated scanner (UMAX Powerlook III; Dallas, TX) at 300 dpi using the same settings for all gels. Flour proteins were identified previously by MS/MS [13] and additional proteins in the endosperm were identified by the same methods. Progenesis SameSpots Ver. 4.0 (Nonlinear Dynamics Limited, Newcastle upon Tyne, UK) was used to detect spots, match spot patterns, normalize and quantify spot volumes, and create protein accumulation profiles. The means and standard deviations for individual spot volumes were calculated and found to be quite similar among the replicate gels for each time point. All of the gluten proteins had Anova p-values less than 0.05 and Power greater than 0.8. Progenesis SameSpots was also used to analyze 2-D gel patterns by principal component analysis and match protein accumulation patterns by correlation analysis. The spot volume data were analyzed by hierarchical cluster analysis using the TIGR MultiExperimentViewer (MeV) v. 4.0 ([50], http://www.tm4.org/mev/]).

## Abbreviations

2-DE: Two-dimensional gel electrophoresis; dpa: Days post-anthesis; HMW-GS: High molecular weight-glutenin subunits; LMW-GS: Low molecular weight-glutenin subunits; LMW-GSA: Large group of LMW-GS; LMW-GSB: Small group of LMW-GS; MS/MS: Tandem mass spectrometry

## Competing interests

The authors declare that they have no competing interests.

## Authors’ contributions

WH was responsible for data analysis and interpretation and drafted the manuscript; CT carried out the quantitative 2-DE gel analysis and spot digestions; WV was responsible for mass spectrometry analysis and database management; RT contributed to data analysis and interpretation and manuscript editing; SA contributed to design of plant growth conditions, data analysis and interpretation, and manuscript editing. All authors read and approved the final manuscript.

## Supplementary Material

Additional file 1 Representative 2-D gels of endosperm proteins collected at specified time points for each of the growth regimens. Click here for file

Additional file 2 Mass spectrometry data for newly identified wheat endosperm proteins. Click here for file

Additional file 3 **Normalized spot volume data for proteins extracted from developing wheat endosperm and separated by 2-DE. The data are listed in the order shown in Figure 3.** MN, MF, HN, and HF are defined in Table 1. Click here for file

Additional file 4 **Normalized spot volume data for proteins extracted from wheat flour and separated by 2-DE.** The data are listed in the order shown in Table 2. MN, MF, HN, and HF are defined in Table 1. Click here for file
