# Supplementary material for: Comparative proteomic analysis of the effect of temperature and fertilizer on gliadin and glutenin accumulation in the developing endosperm and flour from Triticum aestivum L. cv. Butte 86
Source: Proteome Sci. 2013 Feb 22;11:8. doi: 10.1186/1477-5956-11-8 (PMC3599944; doi:10.1186/1477-5956-11-8)

Additional file 1A: Representative 2-D gels of endosperm proteins collected from 8 to 37 dpa under a moderate temperature regimen without fertilizer.

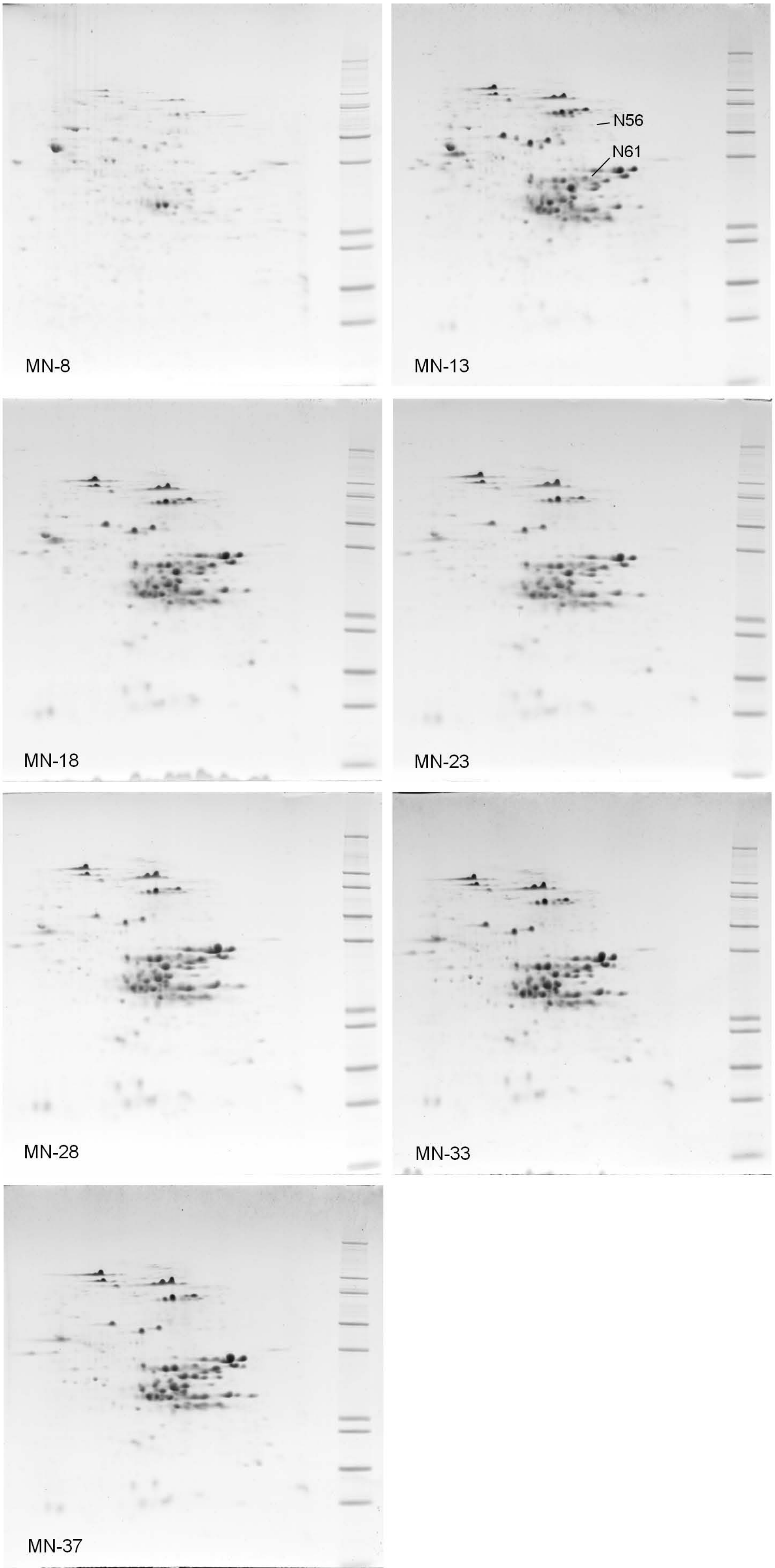

Additional file 1B: Representative 2-D gels of endosperm proteins collected from 8 to 37 dpa under a moderate temperature regimen with fertilizer.

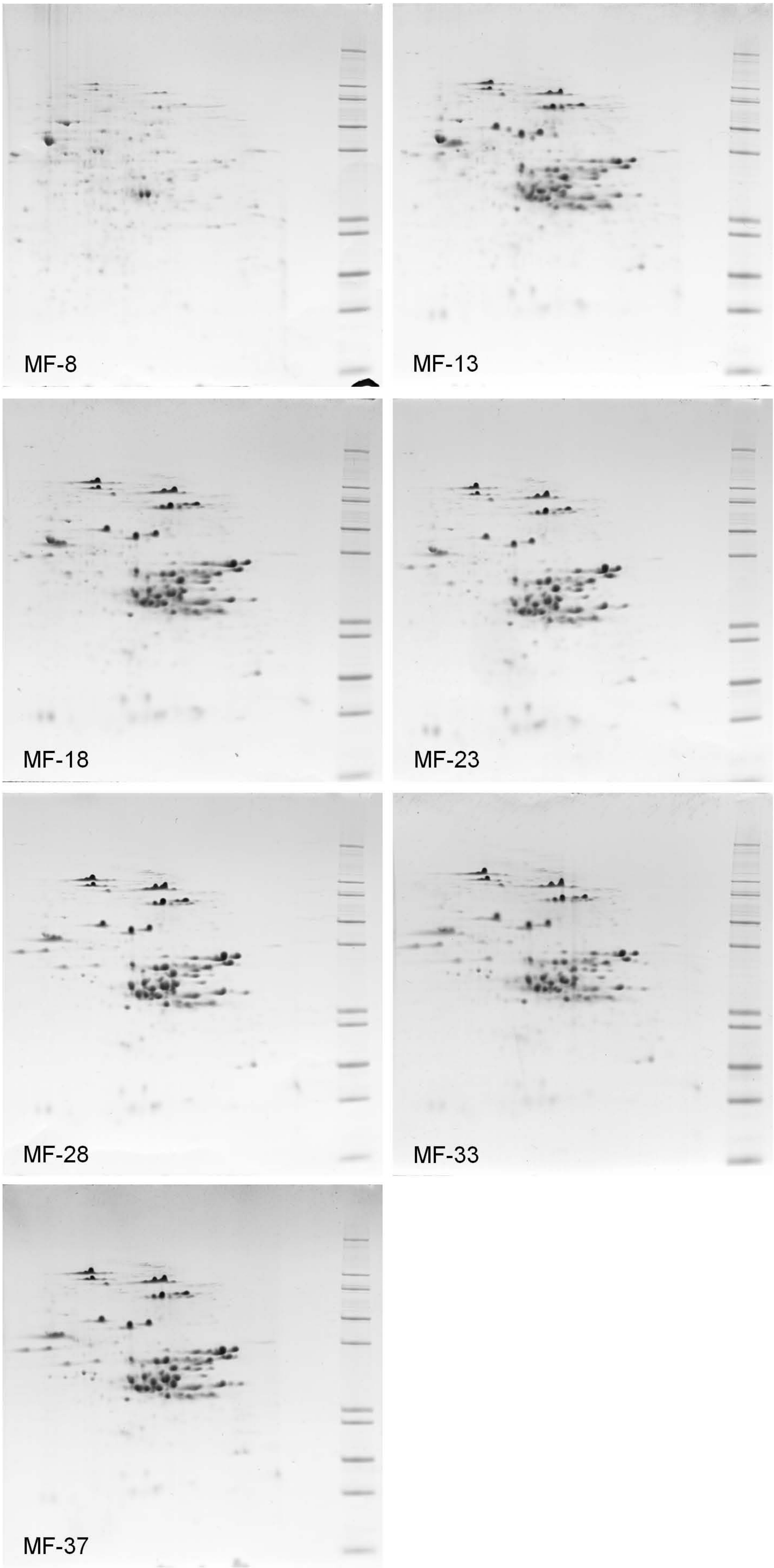

Additional file 1C: Representative 2-D gels of endosperm proteins collected from 6 to 21 dpa under a high temperature regimen without fertilizer.

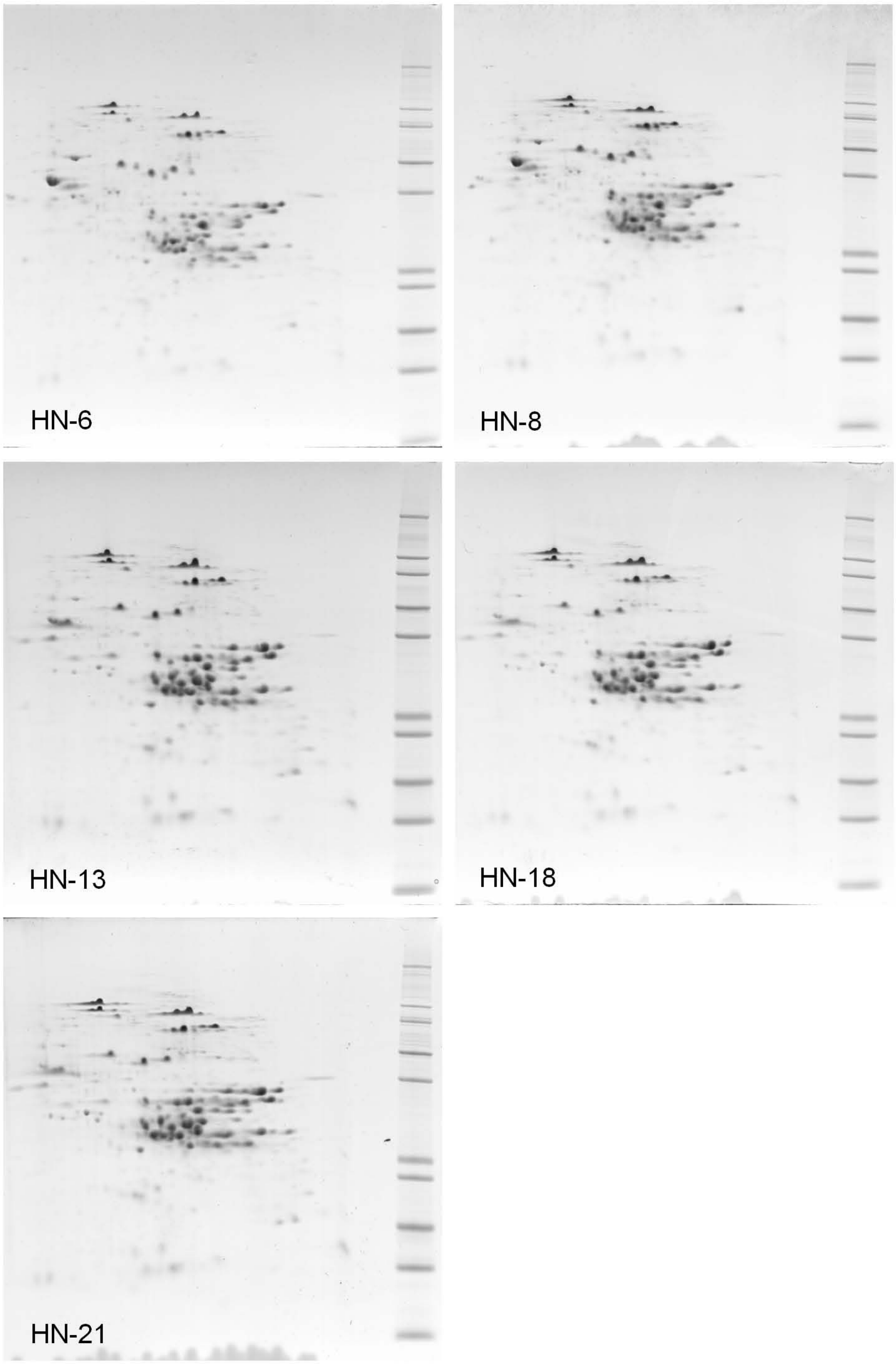

Additional file 1D: Representative 2-D gels of endosperm proteins collected from 6 to 21 dpa under a high temperature regimen with fertilizer.

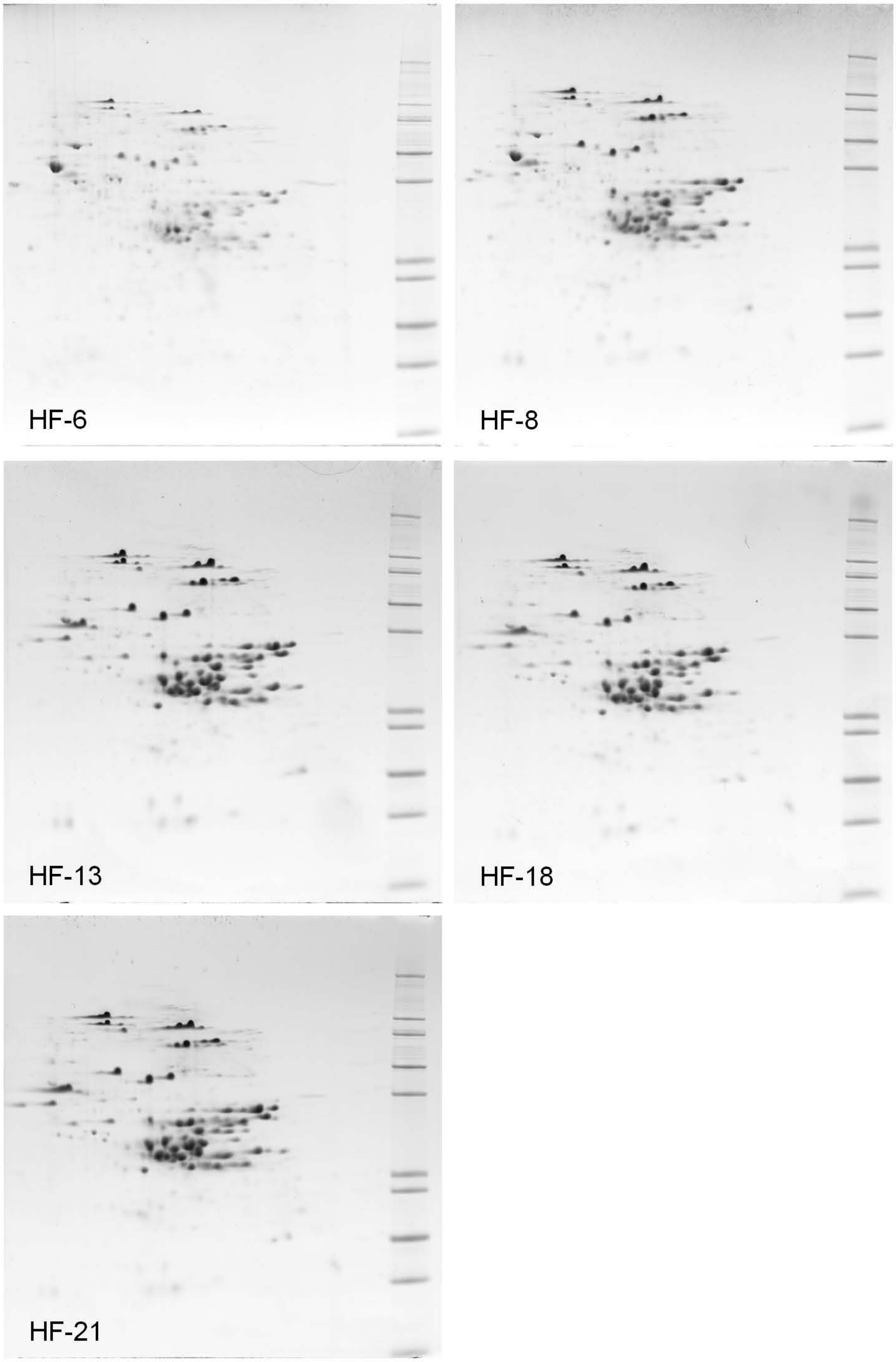

Supplement: Additional file 1 — Representative 2-D gels of endosperm proteins collected at specified time points for each of the growth regimens. [file 1477-5956-11-8-S1.pdf]
